# Oral zinc supplementation restore high molecular weight seminal zinc binding protein to normal value in Iraqi infertile men

**DOI:** 10.1186/1471-2490-12-32

**Published:** 2012-11-13

**Authors:** Mahmoud Hussein Hadwan, Lamia A Almashhedy, Abdul Razzaq S Alsalman

**Affiliations:** 1 Chemistry Department, College of Science, Babylon University, Hillah, Iraq; 2 Surgery Department, College of Medicine, Babylon University, Hillah, Iraq

**Keywords:** Zinc, Zinc binding protein, Gel filtration, Asthenozoospermia, Semenogelin

## Abstract

**Background:**

Zinc in human seminal plasma is divided into three types of ligands which are high (HMW), intermediate (IMW), and low molecular weight ligands (LMW). The present study was aimed to study the effect of Zn supplementation on the quantitative and qualitative characteristics of semen along with Zinc Binding Protein levels in the seminal plasma in asthenozoospermic patients.

**Methods:**

Semen samples were obtained from 37 fertile and 37 asthenozoospermic infertile men with matched age. The subfertile group was treated with zinc sulfate, every participant took two capsules per day for three months (each one 220mg). Semen samples were obtained (before and after zinc sulfate supplementation). After liquefaction seminal fluid at room temperature, routine semen analyses were performed. For determination of the amount of zinc binding proteins, the gel filtration of seminal plasma on Sephadex G-75 was performed. All the fractions were investigated for protein and for zinc concentration by atomic absorption spectrophotometry. Evaluation of chromatograms was made directly from the zinc concentration in each fraction.

**Results:**

A significant high molecular weight zinc binding ligands percentage (HMW-Zn %) was observed in seminal plasma of fertile males compared with subfertile males. However, seminal low molecular weight ligands (LMW-Zn) have opposite behavior. The mean value of semen volume, progressive sperm motility percentage and total normal sperm count were increased after zinc sulfate supplementation.

**Conclusions:**

Zinc supplementation restores HMW-Zn% in seminal plasma of asthenozoospermic subjects to normal value. Zinc supplementation elevates LMW-Zn% in seminal plasma of asthenozoospermic subjects to more than normal value.

**Trial registration:**

ClinicalTrials.gov identifier NCT01612403

## Background

Infertility is defined as lack of ability to conceive within one year of unprotected intercourse with the same partner [1]. It is estimated that nearly 8‐12% of couples are infertile [2]. There are several causes leading to male infertility, like radiation, cigarette smoking, varicocele, antibacterial drugs, infections, obstructive lesions, therapeutic drugs, trauma, genitourinary infection, environmental agents, oxidative stress, and nutritional deficiency of trace elements like, selenium and zinc [3,4]. Zn is the following only to iron as the most abundant element in the body. Although, Zinc is found in red meat, white meat, fish, and milk; the World Health Organization (WHO) approximates that one-third of world population is deficient in Zn [5]. Zinc and citrate are excreted from the prostate gland as a low molecular weight complex, for that explanation, it is estimated that zinc levels in seminal plasma typically represent the prostatic secretory function. After ejaculation, halve quantity of this complex is redistributed and linkage to medium and high molecular weight compounds which generated from the seminal vesicles [6]. Zn is vital to reproductive potential. It has been reported to protect sperm from bacteria and chromosomes damage [7]. Also, it plays a central role in normal testicular growth, spermatogenesis, and sperm physiology [8]; it conserves genomic integrity in the sperm and stabilizes connection of sperm head to tail [9]. Deficiency of Zn is associated with hypogonadism and insufficient growth of secondary sex characteristics in human beings [10]. Low seminal Zn levels were coupled with a decrease in fertilizing ability of sperm [11] and decreased the synthesis of testosterone [12,13]. Also, it can cause atrophy of the seminiferous tubules in the rat, and that leads to malfunction in spermatogenesis and impotence [14].

The binding of zinc with some proteins, such as metallothioneins [MT] and α-2 macroglobulin [α-2M] is fundamental immune efficiency for the duration of ageing and in age-related diseases. These proteins may turn from a function of protection against cellular oxidative injury. Furthermore, zinc-binding proteins regain their essential role of cellular protection against oxidative damage after zinc supplementation [15].

Studies have shown that oral zinc supplementation develops sperm count, motility and the physical characteristics of sperm in animals [16,17] and also, in some groups of infertile men [18,19]. The present study was conducted to study the effect of Zn supplementation on the quantitative and qualitative characteristics of semen along with Zinc Binding Protein levels in the seminal plasma of asthenozoospermic patients.

## Methods

### Patients

This study includes 37 subfertile male partners between July 2011 to April 2012, from couples who had consulted the infertility clinic of the Babil hospital of maternity (Hilla city/IRAQ). The approval of the institutional research ethics committee, and consent of every patient included in the study was obtained. A detailed medical history was taken and physical examination was performed. Subjects currently on any medication or antioxidant supplementation were not included. The inclusion criteria were asthenozoospermia, the absence of endocrinopathy, varicocele, and female factor infertility. Smokers and alcoholic men were excluded from the study because of their recognized high seminal ROS levels and decreased antioxidant levels. The selection criteria of fertile group were the absence of asthenozoospermia, endocrinopathy, varicocele, and have a birth in the last year.

### Semen analysis

Semen samples were obtained from 37 fertile (age 31.4±4.2 year) and 37 subfertile (age 32.3±3.68 year) men with asthenozoospermia by masturbation after three days of sexual abstinence. The subfertile group was treated with zinc sulfate, every participant took two capsules of zinc sulfate per day for three months (each one 220mg). Semen samples were obtained (before and after zinc sulfate supplementation). After liquefaction seminal fluid at room temperature, routine semen analyses including semen volume, pH, concentration, sperm motility, normal sperm morphology and round cell were performed according to 2010 WHO recommendation [20].

An aliquot of the remaining semen was centrifuged at 2000 g for 15 min and the seminal plasma was collected immediately. These fractions were classified into three groups called group I (healthy donors), group II (patients before treatment) and group III (patients after treatment) respectively. After that, the samples were frozen (−20°C) until analyzed.

### Chemicals

All reagents and chemicals were of analytical grade and obtained from standard commercial suppliers.

### Biochemical procedures

#### Gel filtration

For determination of the amount of zinc binding proteins, the gel filtration of seminal plasma on Sephadex G-75 was performed according to Arver method exactly [21]. Sephadex G-75 was packed in a 2.5×40 cm glass column and equilibrated and eluted with 0.05 M Tris buffer containing 0.15 M NaCl, pH 7.4. The elution was collected at a flow rate of 10–16 ml cm ^–2^ h ^–1^ in 50 fractions. All the fractions were investigated for protein (A 280nm) and for zinc concentration by atomic absorption spectrophotometry (shimadzu AA 6300). Homogeneity of gel packing and void volume (V_**o**_) was checked with Dextrane Blue 2000 (Pharmacia, Sweden). Total column volume was calculated from column dimensions. Evaluation of chromatograms were made directly from the zinc concentration in each fraction and the peaks were referred to as I, II, and III or HMW, IMW and LMW (High, Intermediate and Low molecular Weight zinc binding protein) respectively. The area of peak was calculated by Simpson’s 1/3 rule [22].

### Statistical analysis

Student’s *t* and the Mann–Whitney *U*-tests were used for statistical analyses.

### Ethical committee

Iraq: Ethics Committee

## Results

The results in Table ( 1) indicate the baseline, characteristics of the semen parameters are depicted in the fertile and subfertile (before and after treatment with zinc sulfate) groups. These parameters were significantly decreased in infertile group compared with healthy donors group. However, the level of the semen parameters were significantly increased (return to normal value) after zinc sulfate supplementation.

**Table 1:** Ejaculates parameters

	**Semen volume (ml)**	**Sperm count (×10**^**6**^**)**	**Progressive sperm motility (%)**	**Normal sperm form (%)**
**Healthy donors**	2.8±0.43	83±16	68±12	77±5
**Patients before treatment**	1.72±0.66*	68±17*	22±7*	66±12
**Patients after treatment**	2.38±0.6**	70±17	38±8**	71±8**

Sn1*: significance versus group I (Healthy donors).

Sn2 **: significance versus group II (Patients before treatment).

**Figure 1:**
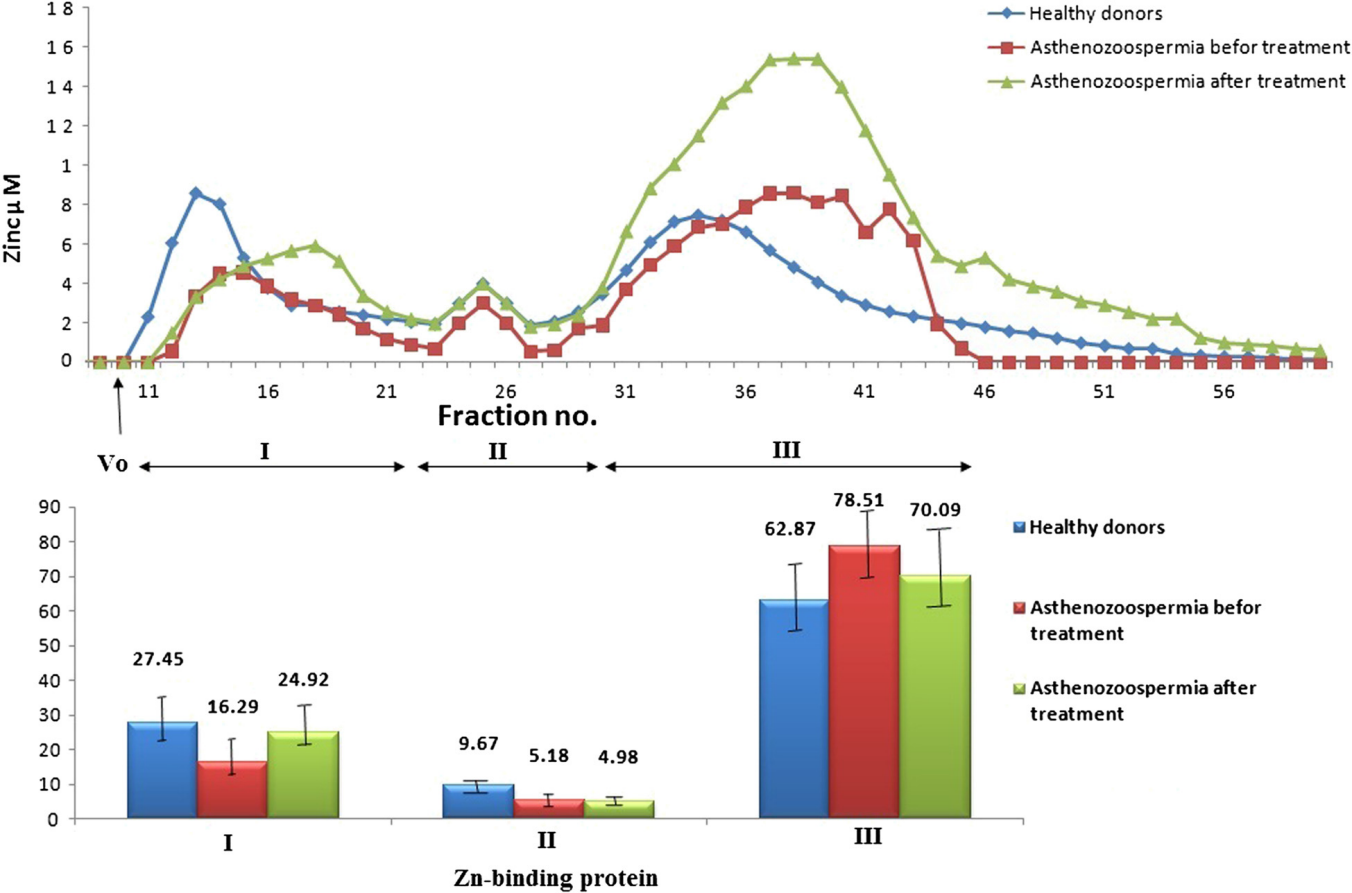
**Above: Distribution of zinc in one sample of human seminal plasma of each group (group I (healthy donors), group II (patients before treatment) and group III (patients after treatment)) subjected to gel filtration on Sephadex G-75.** Below: The combined results from 9 semen samples of each group subjected to gel filtration as shown above.

The common appearance of the zinc distribution between different ligands in human seminal plasma as discovered by gel filtration on Sephadex G-75 is demonstrated in Figure 1. The HMW (peak I) and LMW (peak III) ligands have characteristic chromatographic properties but the IMW ligands (peak II) show an inconsistent pattern [21]. 

We observed a significant higher semen high molecular weight zinc binding ligands percentage (HMW-Zn %) in fertile males compared with subfertile males; however, zinc supplementation restores this percentage in the subfertile men to the normal ranges. On the other hand, seminal low molecular weight ligands (LMW-Zn) have opposite behavior. IMW- Zn% decreased significantly in semen of patients of asthenozoospermia.

## Discussion

The concentration of zinc in human seminal plasma is higher than blood and tissues [23]. Zinc is part of copper -zinc superoxide dismutase and several proteins involved in the cell differentiation (eg, glycoprotein IIb/IIIa) as well as the factors activation such as KS-1, WT-1, Finb, TRAF-2, and ZEB [24-26]. Zinc deficiency affects reproduction adversely in view of the fact that all the hormones and a wide range of enzymes involved in reproduction are receptive to zinc. Principally, zinc is required for the synthesis and secretion of luteinizing hormones and follicle-stimulating hormone [27].

There have been conflicting clinical reports on the effect of seminal zinc on sperm count, motility and the physical characteristics of sperm. Several studies have pointed out that there are no significant difference between Zn levels in fertile and infertile men [28-31] but furthers found a significant difference between them [32-34]. Danscher *et al.*[35] indicated a high concentration of Zn to be linked with poor motility of sperm. Also, Carpino *et al*. [36] have reported high sperm zinc content to be associated with oligoasthenozoospermic specimens. On the other hand, others have observed a high zinc content in seminal plasma to be correlated with good physical characteristics of sperm such as sperm count [31,32], motility [33,37], and normal morphology [33,38].

As a result, total seminal zinc concentration may not be a useful indicator of the zinc fraction associated with physical characteristics of sperm and a more appropriate marker of the ion bioavailability should be utilized to assess its relationship to sperm functions. The best proof is zinc binding protein because all the previous studies as well as this study observed that zinc binding protein correlated with sperm functions such as count and motility [28,33].

Seminal zinc has been investigated by measuring the amount of zinc bound to vesicular high, intermediate and low molecular weight protein. HMW-Zn% was decreased in asthenozoospermic subjects. While, low zinc binding protein LMW-Zn% exhibits reverse behavior, so it decreased in healthy fertile men and increased in subfertile.

The mechanism by which HMW-Zn% in seminal plasma of asthenozoospermic subjects is depleted has not been fully explained in previous studies. Different studies have shown that Zn has an important role in scavenging reactive oxygen species (ROS) owing to it has antioxidative properties [39-41]. Elevated levels of ROS were detected in the semen of high percentage of infertile patients, which affected sperm function [42]. As a result, to this elevation of ROS levels, Zn levels may be reduced in seminal plasma of asthenozoospermic subjects, and that leads to an increase in oxidation of HMW-Zn binding proteins in seminal plasma. The last processes are associated with abnormal sperm parameters. Zinc supplementation restores HMW-Zn% in seminal plasma of asthenozoospermic subjects to normal value, may be because its role in polymeric organization of macromolecules likes protein synthesis and cell division [43] or because its ability to influence the process of spermatogenesis and maintains the ability of sperm nuclear chromatin to undergo de-condensation and modulates sperm functions [44].

**Figure 2:**
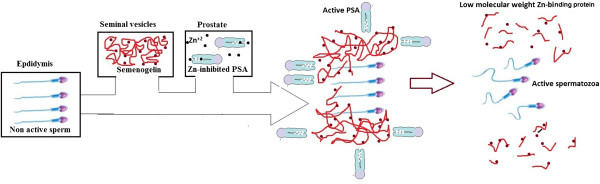
The coagulation and liquefaction of human semen.

LMW-Zn% is elevated in seminal plasma of asthenozoospermic patients may be because increment the levels of semenogelin in seminal plasma of asthenozoospermic subjects. Martinez-Heredia *et al.*[45] used 2DE-MS to recognize 17 protein spots with differential expression levels between asthenozoospermic samples and controls. Markedly, semenogelin has been shown to elevate in asthenozoospermic samples. Zhao *et al.*[46] demonstrated that semenogelin I precursor is recognized as four kinds of protein. Two of these proteins characterized 14 kDa fragments and the other two were 17 kDa fragments. All of these proteins were highly expressed in asthenozoospermic patients. Before this scientific finding, Yoshida *et al.*[47] documented that a 14 kDa fragment of semenogelin has an inhibitory consequence on ejaculated spermatozoa. Semenogelin is secreted from the seminal vesicle at ejaculation and establish major structural components of coagulated human semen. It is defined as a sperm motility inhibitor, which is classified into two types of intermediate molecular weight zinc binding protein (IMW-ZnBP), [because it has molecular weight more than 3000 and less than 80 000], the first Semenogelin I (a protein of molecular weight (MW) 52 kDa) and Semenogelin II (existed as two forms of a Sg I-related protein with MWs of 71 and 76 kDa) [48,49]. After ejaculation, serine proteases, primarily prostate-specific antigen (PSA) act to cleave SgI and SgII molecules (intermediate molecular weight zinc binding protein IMW-ZnBP) to produce soluble fragments (Low molecular weight zinc binding protein LMW-ZnBP) [50]. PSA is accumulated in the prostate in a Zn^2+^ -inhibited type. However, PSA is activated after mixing with Semenogelin, which has a higher Zn^2+^ -binding affinity than PSA [51]. In corresponding to this liquefaction, the spermatozoa become regularly more motile, as shown in Figure 2. 

Zinc supplementation elevates LMW-Zn% in seminal plasma of asthenozoospermic subjects to more than normal value, that’s may be because its enhancement the synthesis of metallothioneins (Low molecular weight zinc binding protein) [52,53]. The increment of LMW-Zn% not coming from the degradation of semenogelin because Zn^2+^ acts to inhibit the protease activity of prostate-specific antigen [54]. The increment of LMW-Zn% after supplementation is not harmful to spermatozoa because it’s not generating from the degradation of semenogelin, i.e. it’s not contain 14 kDa fragment of semenogelin which has an inhibitory effect on ejaculated spermatozoa.

## Conclusions

Zinc supplementation restores HMW-Zn% in seminal plasma of asthenozoospermic subjects to normal value. Zinc supplementation elevates LMW-Zn% in seminal plasma of asthenozoospermic subjects to more than normal value.

## Abbreviations

ROS: Reactive Oxygen Species; SOD: Superoxide Dismutase; Zn: Zinc; AAS: Atomic Absorption Spectroscopy; WHO: World Health Organization; HMW: High molecular weight zinc binding protein; IMW: Intermediate molecular weight zinc binding protein; LMW: Low molecular weight zinc binding protein.

## Competing interests

The authors declare that they have no competing interests.

## Authors’ contributions

All the authors made important roles to the design and viewing of the study. Principally, MHH wrote the manuscript, contributed to the investigation and elucidation of the data. LAA participated in its design and coordination and assisted to draft the manuscript. ARA contributed to the implementation of the protocol. All the authors have been involved in drafting and revising the manuscript, have read, and approved the final manuscript.

## Pre-publication history

The pre-publication history for this paper can be accessed here:

http://www.biomedcentral.com/1471-2490/12/32/prepub
